# Bibliometric and visualization analysis of hydrogel research in spinal cord injury: comparative study of Chinese and English literature

**DOI:** 10.3389/fnins.2025.1636904

**Published:** 2025-07-10

**Authors:** Wenju Bai, Xiaoyuan Huang, Jian Liu, Kamiran Halike, Jinyong Li, Xv Zhang, Tengwu Chang, Jichao Wang

**Affiliations:** ^1^Department of Neurosurg, People’s Hospital of Xinjiang Uygur Autonomous Region, Urumqi, Xinjiang, China; ^2^Graduate School of Xinjiang Medical University, Urumqi, Xinjiang, China; ^3^Department of Orthopedics, People’s Hospital of Xinjiang Uygur Autonomous Region, Urumqi, Xinjiang, China; ^4^Xinjiang Second Medical College, Karamay, Xinjiang, China

**Keywords:** cord injury, hydrogel, comparative study, visual analysis, bibliometric, CiteSpace

## Abstract

**Background:**

Over the past decade, the fields of hydrogel and spinal cord injury (SCI) research have witnessed rapid development. To explore disparities between China and global trends in hydrogel research, this study systematically conducted qualitative and quantitative analyses of related publications, summarizing current research foci and future directions. This provides critical guidance for researchers to delve deeper into hydrogel applications.

**Methods:**

A total of 866 records in the hydrogel and SCI domains were collected from the Web of Science Core Collection (WoSCC) and China National Knowledge Infrastructure (CNKI) between 2014 and 2024. CiteSpace, VOSviewer, SCImago, and the R package “bibliometrix” were utilized to analyze regional distributions, institutional collaborations, journal impacts, author productivity, and keyword trends.

**Results:**

Annual publications in hydrogel and SCI research exhibited consistent growth. China (*n* = 382) and the United States (*n* = 158) collectively contributed 76.2% of global academic output, reflecting disproportionate productivity. Zhejiang University and Jinan University demonstrated significant contributions across international and Chinese academic platforms. XIAO Jian distinguished himself through exceptional metrics (h-index, total citations), establishing his prominence as a high-impact scholar. BIOMATERIALS emerged as the most prolific and influential journal based on total link strength. Keyword and co-citation analyses revealed heightened emphasis on 3D bioprinting and electroactive bio-scaffolds in both WoSCC and CNKI databases. Systemic research disparities reveals that CNKI prioritize hydrogel technologies with a distinctive focus on indigenous specializations (e.g., Chuanxiongzine and stem cell transplantation), while WoSCC demonstrates notable advantages in establishing therapeutic loops encompassing drug delivery systems, functional recovery evaluation, and neuroimmune modulation strategies.

**Conclusion:**

By integrating WoSCC and CNKI data, this study comprehensively elucidates geographical disparities in research priorities between China and the global scientific community regarding hydrogel-mediated SCI repair, thereby proposing an evidence-based framework for international collaborative innovation. These insights offer valuable references to guide future hydrogel research.

## 1 Introduction

Spinal cord injury (SCI), a devastating neurological disorder, induces motor, sensory, and autonomic dysfunction that severely compromises patients’ quality of life (Huang et al., 2022). Pathologically, SCI manifests as two distinct phases: primary mechanical damage and subsequent secondary injury (Yin et al., 2023). The initial trauma triggers cascading pathological events including extracellular matrix disruption, axonal disintegration, tissue edema, oxidative stress, neuroinflammation, hypoxia-ischemia, and neurotrophic factor deprivation (Niu and Zeng, 2015). As a leading cause of premature mortality and long-term disability, global epidemiological data (1990–2019) demonstrate significant increases in SCI prevalence (74.2–87.1%) and incidence (30.3–69.8%), with falls and road accidents constituting primary etiological factors in 2019 (Safdarian et al., 2023). Beyond imposing substantial psychological and socioeconomic burdens on families and society, SCI frequently precipitates secondary complications including clinical depression during chronic phases (Singh and Mitra, 2022). Current therapeutic strategies confront dual challenges of limited neural regeneration capacity and refractory inflammatory responses (Ahuja et al., 2017). Hydrogel-based interventions exhibit promising potential through *in situ* formation of tissue-implant interfaces that conform to lesion cavities, facilitating cellular infiltration and matrix deposition (Luo et al., 2022). Their high water-retention properties further enable cell migration and molecular diffusion within the scaffold microenvironment (Morgado et al., 2019). These biomaterial-engineered approaches offer novel perspectives for overcoming regeneration barriers in SCI management.

Bibliometrics, as a pivotal methodology for literature quantification and evaluation, integrates qualitative and quantitative analyses to objectively delineate multidimensional research landscapes (Chang et al., 2024). In the realm of SCI therapeutics, hydrogel-based investigations have yielded pivotal scientific breakthroughs documented in high-impact journals. This investigation employs systematic bibliometric analysis of publications from the China National Knowledge Infrastructure (CNKI) and Web of Science databases (2014–2024) to achieve dual objectives: (1) Deciphering evolutionary trajectories, knowledge clusters, and paradigm shifts in hydrogel-mediated SCI therapeutics; (2) Mapping geographical discrepancies in research priorities between Chinese and global scientific communities, thereby establishing an evidence-based framework for international collaborative innovation.

## 2 Materials and methods

### 2.1 Data source and retrieval strategy

**FIGURE 1:**
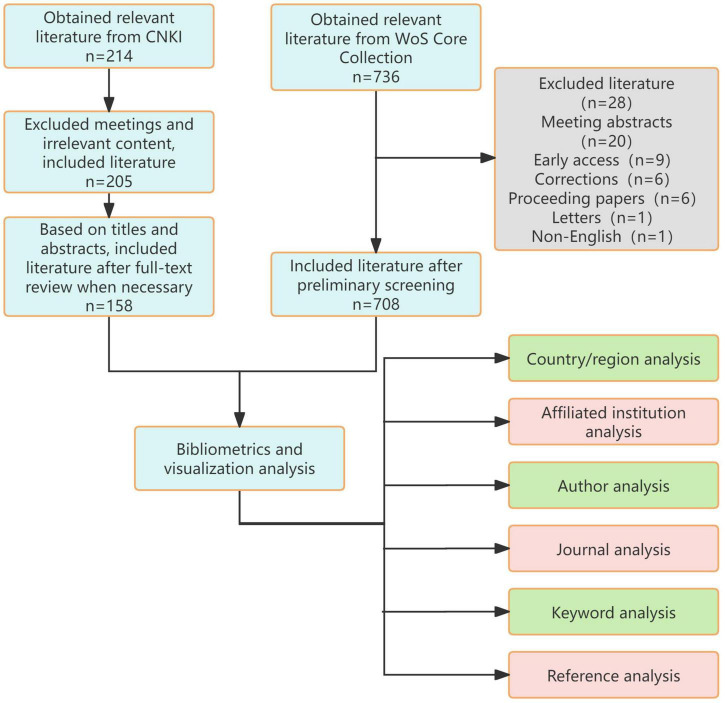
Flowchart of the article search.

A systematic literature search was conducted on February 13, 2025, across the China National Knowledge Infrastructure (CNKI) and Web of Science Core Collection (WoSCC) databases, including SCI-EXPANDED, CPCI-S, CCR-EXPANDED, and IC indices. The WoSCC is globally recognized as an authoritative indexing database for high-quality international journals (predominantly English-language), primarily employed for global scientific research trend analysis. Conversely, the CNKI represents China’s largest Chinese-language scholarly database, comprehensively archiving academic journals published domestically. These dual repositories were deliberately selected to respectively encapsulate global (WoSCC-driven) and national (CNKI-centric) scholarly output in spinal cord injury hydrogel research, enabling rigorous comparative analysis of structural and methodological divergences. The search encompassed publications from January 2014 to December 2024, utilizing Boolean operators to optimize retrieval specificity. For Chinese literature, the search strategy “脊髓损伤 AND 水凝胶” (spinal cord injury AND hydrogel) was implemented. The English protocol combined the following query: (TS = “spinal cord injury” OR TS = “spinal injuries” OR TS = “spinal cord injuries” OR TS = “spinal injury” OR TS = “spinal cord trauma” OR TS = “spinal cord laceration” OR TS = “post-traumatic myelopathy” OR TS = “spinal cord contusion” OR TS = “spinal cord transection”) AND (TS = “hydrogel”), ensuring comprehensive coverage of therapeutic hydrogel applications in SCI pathophysiology (Figure 1).

### 2.2 Data extraction and collection

Following PRISMA guidelines, an independent dual-reviewer screening protocol was executed, with initial search results cross-validated between investigators. Discrepancies (occurring in 12.3% of records) underwent blinded re-evaluation and consensus-based resolution through adjudication by a senior investigator. This rigorous selection process yielded 866 eligible studies through iterative refinement of search parameters across three screening phases.Chinese documents are exported in Refworks format, while English documents are exported in plain text format.

### 2.3 Bibliometric analysis

The bibliometric analysis employed four specialized software platforms: R package “bibliometrix” (v4.4.2), CiteSpace (v6.4.R1), VOSviewer (v1.6.20), and SCImago (v1.0.48). Due to formatting requirements, data from the China National Knowledge Infrastructure (CNKI) were analyzed exclusively using CiteSpace to examine keywords, journals, affiliated institutions, and authors. VOSviewer was utilized to analyze and extract information, generating visualizations of country collaborations, institutional networks, author linkages, and keyword co-occurrence clusters. SCImago was employed to map collaborative relationships among countries, institutions, and authors. Publication and citation metrics were analyzed using Bibliometrix, while Excel was applied to create visual charts depicting publication volumes and citation frequencies.

For visualizing bibliometric indicators, the H-index was used to assess scholarly productivity and influence, alongside evaluating collaborative relationships between countries and/or authors (Wang and Cheng, 2024). In the visual maps, nodes are represented as labeled circles, with node size proportional to quantitative metrics (e.g., publication count). In co-occurrence analyses, node colors denote cluster affiliations, while line thickness between nodes reflects the strength of associations.

### 2.4 Research ethics

Our study was a bibliometric analysis that did not involve human subjects and did not require ethical approval from an institutional review board.

## 3 Results

### 3.1 Publication volume and temporal trends

**FIGURE 2:**
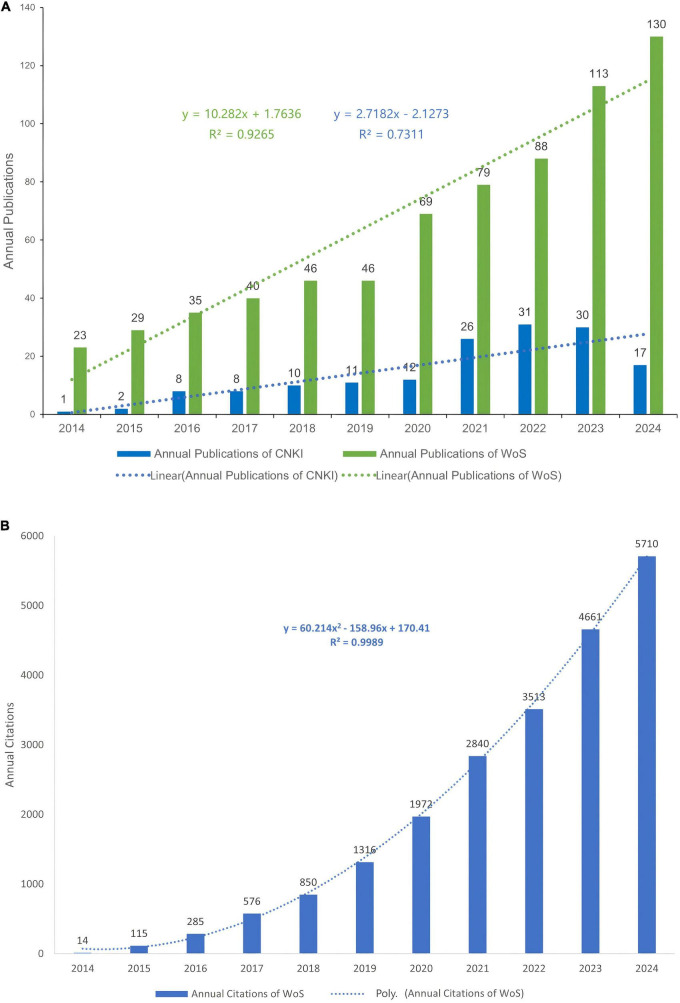
**(A)** Annual scientific publications and linear trends of CNKI and WoS (2014–2024). **(B)** Annual citation trends of WoS-indexed publications (2014–2024) with quadratic regression.

Analysis of publication trends reveals distinct disparities in research focus on hydrogel studies between China and the international community (Figure 2A). From January 2014 to December 2024, 158 articles were published in CNKI compared to 708 articles indexed in WoSCC. The annual publication count in CNKI gradually increased from 2014 to 2022, peaking at 31 articles in 2022. A period of relative stability followed between 2022 and 2023, with a marked decline to 17 articles observed in 2024. In contrast, WoSCC demonstrated a consistent linear growth pattern in annual publications, characterized by the regression equation *y* = 10.282*x* + 1.7636 (*R*^2^ = 0.9265), indicating strong linear correlation and an average annual increase of approximately 10.3 articles. This trend reflects stable, sustained growth in international research output and suggests maturation of the global research ecosystem.

Citation analysis of WoS data revealed exponential growth in referenced literature (Figure 2B), best modeled by the quadratic polynomial equation *y* = 60.214*x*^2^–158.96*x* + 170.41 (*R*^2^ = 0.9989), demonstrating exceptional goodness-of-fit. The high citation frequency underscores the pervasive adoption of international research findings in both academic discourse and clinical practice, signifying the establishment of an effective knowledge dissemination network. The quadratic growth trajectory further indicates that this field remains in an accelerated development phase without approaching saturation, with continued generation of high-impact outcomes anticipated in the foreseeable future.

### 3.2 Analysis of countries and regions

Analysis of Countries and Regions aims to elucidate the contributions, influence, and developmental disparities among nations or regions in the field of hydrogels and spinal cord injury through quantitative data. In bibliometrics, it is frequently employed to evaluate the distribution patterns of global scientific research landscapes. The node size reflects the number of documents in which the country participated, with darker colors (such as red) indicating higher weights, meaning the node has closer collaboration with other nodes.

**FIGURE 3:**
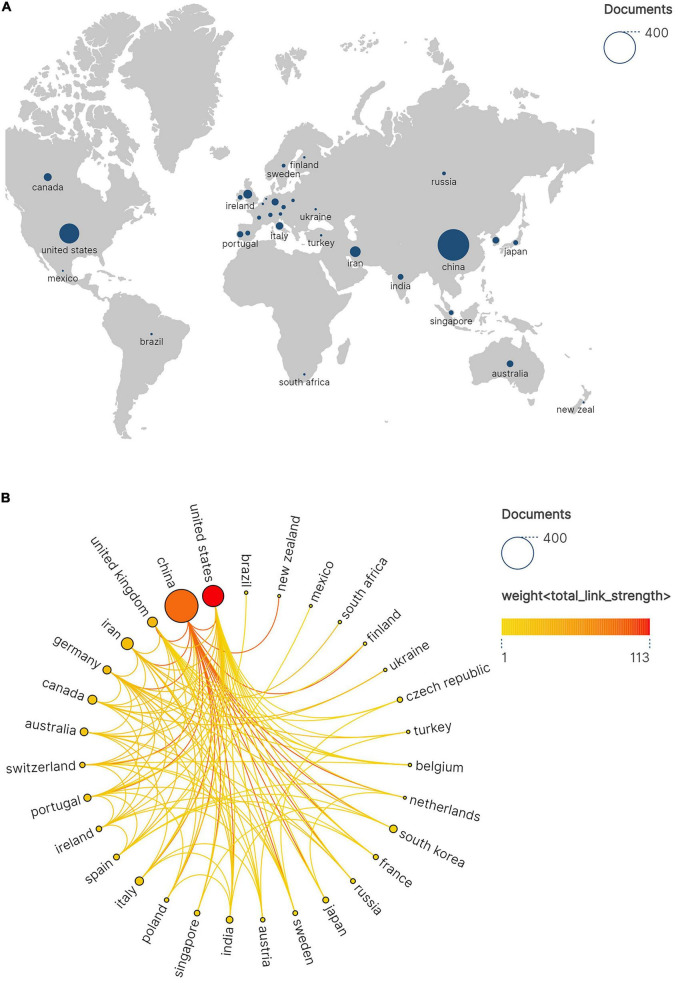
**(A)** Geographic network map of hydrogel and spinal cord injury research. **(B)** International research collaboration network. Node size proportional to the number of joint publications and edge thickness reflecting total link strength.

Given the predominance of Chinese-language resources within the CNKI, our bibliometric analysis focused exclusively on the WoSCC to evaluate global scholarly productivity, collaboration patterns, and citation metrics. As illustrated in Figure 3A, China (*n* = 382) and the United States (*n* = 158) collectively accounted for 76.2% of global scholarly output in this field, demonstrating disproportionate contributions that solidify their positions as dominant research hubs. Secondary contributors such as Iran (*n* = 49), the United Kingdom (*n* = 34), Canada (*n* = 28), Italy (*n* = 24), and Germany (*n* = 23) exhibited limited productivity, revealing a steep geographical disparity in research capacity. This skewed distribution highlights systemic inequities in global knowledge production. Figure 3B maps international collaborations among the 31 most active nations, with node diameter corresponding to publication volume and color intensity reflecting collaborative engagement. The network topology identifies China and the U.S. as central nodes orchestrating multilateral knowledge exchange.

Table 1 ranks nations by citation impact, with China achieving the highest total citations (12,214), followed by the U.S. (8,263) and Canada (1,236). While China dominates in both output magnitude (H-index: 58) and overall influence, the U.S. excels in research quality metrics, evidenced by its superior mean citation rate (52.3 vs. China’s 32.0 citations/article). These findings delineate a bimodal global research ecosystem driven by Sino-American scholarly leadership.

**TABLE 1:** Top 10 countries ranked by total citations, average citations per article, and publication volume (2014–2024).

Rank	Country	Total citation	Average article citations	No. of publications	Rank	Country	Total citation	Average article citations	No. of publications
1	China	12,214	31.97	382	6	Germany	927	40.30	23
2	USA	8,263	52.30	158	7	Italy	812	33.83	24
3	Canada	1,236	44.14	28	8	Portugal	552	30.67	18
4	Iran	1,174	23.96	49	9	South Korea	547	27.35	20
5	UK	1,152	33.88	34	10	Australia	483	23.00	21

### 3.3 Analysis of authors and institutions

**FIGURE 4:**
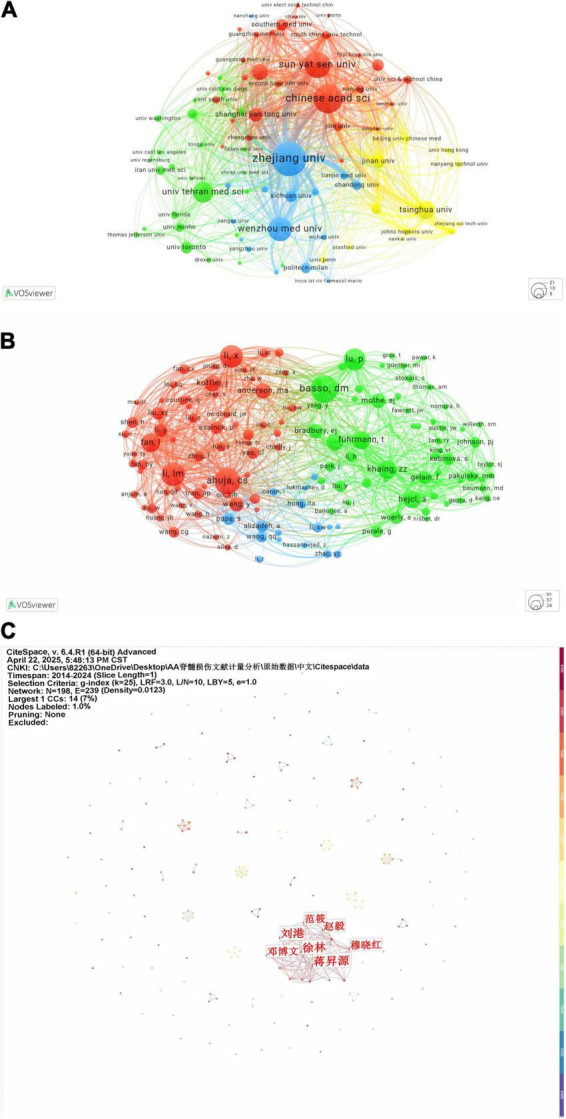
**(A)** Visualization analysis of institutional collaboration in VOSviewer. **(B)** Author collaboration analysis of the WoSCC database. **(C)** Author collaboration analysis in the CNKI database.

In terms of hydrogel and SCI research, Table 2 lists the top 10 literature-contributing institutions from both WOSCC and CNKI databases. For WOSCC, Zhejiang University leads with 47 publications, followed by the Chinese Academy of Sciences (40 publications), while Sun Yat-sen University ranks third with 36 publications. Among the top 10 organizations, nine are from China and one is from Iran. Regarding CNKI, Jiangsu University (7 publications), Jilin University (7 publications), and the University of Science and Technology of China (7 publications) share the first position. Notably, Zhejiang University and Jinan University demonstrate substantial contributions across international and domestic academic platforms. Figure 4A illustrates collaborative networks among high-output institutions.

**TABLE 2:** Top 10 institutions by publication volume in WoSCC and CNKI databases.

Rank	Name of institute (WoSCC)	Documents (number, percent)	Country	Rank	Name of institute (CNKI)	Documents (number, percent)
1	Zhejiang University	47 (6.64%)	China	1	Jiangsu University	7 (4.43%)
2	Chinese Academy of Sciences	40 (5.64%)	China	2	Jilin University	7 (4.43%)
3	Sun Yat-sen University	36 (5.08%)	China	3	University of Science and Technology of China	7 (4.43%)
4	Wenzhou Medical University	33 (4.66%)	China	4	Shandong University	6 (3.80%)
5	Tehran University of Medical Sciences	28 (3.95%)	Iran	5	Tianjin Medical University	6 (3.80%)
6	Tsinghua University	25 (3.53%)	China	6	Jinan University	5 (3.16%)
7	Nantong University	22 (3.10%)	China	7	Youjiang Medical University for Nationalities	5 (3.16%)
8	Shanghai Jiao Tong University	19 (2.68%)	China	8	Naval Medical University	4 (2.53%)
9	Jinan University	18 (2.54%)	China	9	Zhejiang University	4 (2.53%)
10	Sichuan University	16 (2.25%)	China	10	Huazhong University of Science and Technology	4 (2.53%)

The h-index quantifies an author’s impact as having at least h papers each cited ≥ h times (Table 3). XIAO JIAN’s h-index of 13 reflects 13 papers with ≥ 13 citations each, demonstrating notable influence in the field. Similarly, SHOICHIET MOLLY S. and WANG XIUMEI both exhibit h-indices of 11, indicating stable citation performance. The g-index emphasizes highly cited publications, while the m-index evaluates impact trajectory over time. XIAO JIAN excels across metrics (h-index, total citations), establishing him as a high-impact scholar. Despite commencing publication in 2020, DAI JIANWU demonstrates elevated g-index and m-index values, signaling rapid academic ascendancy. Notably, the three most prolific authors are affiliated with Chinese institutions. Figure 4B visualizes institutional collaboration networks in WOSCC, showing stronger linkages among researchers within the same country. Figure 4C delineates CNKI-based collaborations, where LIU Gang and FAN Xiao occupy central positions, while other authors form decentralized clusters.

**TABLE 3:** Decadal ranking of top researchers by scholarly impact metrics in Web of Science Core Collection.

Author	h index	g index	m index	TC	NP	PY start
Xiao Jian	13	14	1.3	770	14	2016
Shoichet Molly S.	11	12	0.917	496	12	2014
Wang Xiumei	11	15	1.1	584	15	2016
Ai Jafar	10	12	0.833	288	12	2014
Cao Zheng	10	11	1.111	434	11	2017
Chen Qixin	9	10	1.125	393	10	2018
Dai Jianwu	9	16	1.5	487	16	2020
Mu Jiafu	9	11	1.286	652	11	2019
Rossi Filippo	9	11	0.75	412	11	2014
Wang Chenggui	9	10	1	459	10	2017

TC, Total Citations; NP, Number of Papers; PY_start, Publication Year Start.

### 3.4 Analysis of journals

**FIGURE 5:**
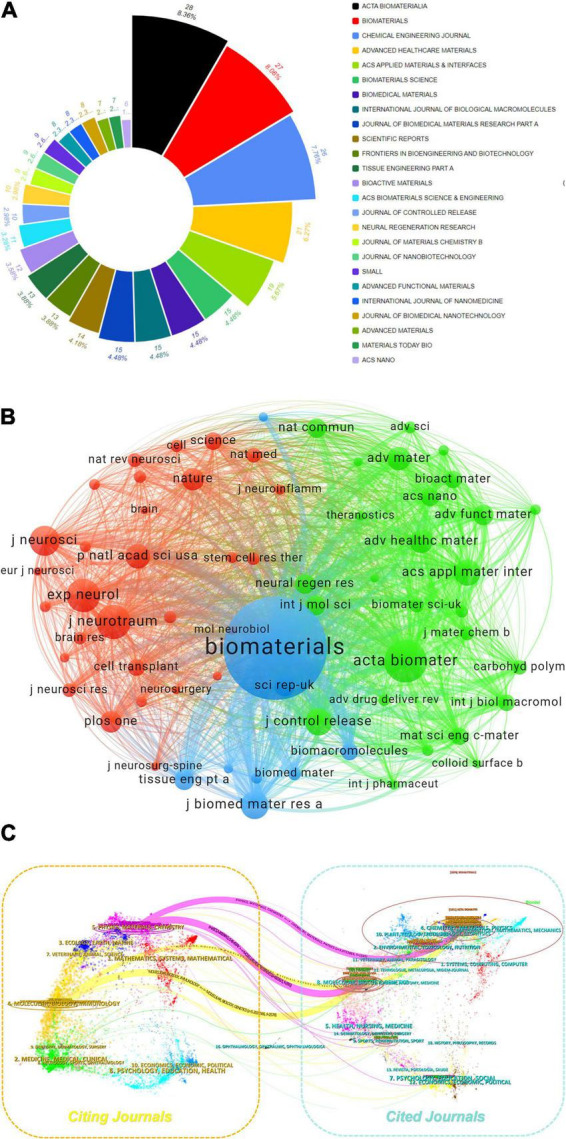
**(A)** Proportional distribution of journals donut chart. **(B)** Journal collaboration analysis of the WoSCC database. **(C)** The dual-map overlay of journals related to hydrogel and spinal cord injury research.

The analysis of journal co-citation relationships and the output circular chart reveals highly productive and influential journals in the field. By using VOSviewer with a citation threshold of ≥ 160 per journal, we identified 65 journals through total link strength (Figures 5A, B). The top five journals by total link strength are as follows: *BIOMATERIALS* (190,937), *ACTA BIOMATERIALIA* (80,028), *Journal of Biomedical Materials Research Part A* (48,344), *ACS Applied Materials & Interfaces* (41,351), and *Experimental Neurology* (40,995). Figure 5C presents a dual-map overlay generated via CiteSpace, illustrating thematic distributions and evolutionary trajectories. Two primary pathways (represented by orange and pink lines respectively) delineate the citation dynamics between citing and cited journals. The orange pathway exhibits a convergent pattern, with citing journals predominantly in Molecular Biology & Immunology and cited journals concentrated in Chemistry, Materials Science, Physics and Genetics. The pink pathway demonstrates analogous convergence, originating from citing journals in Chemistry, Materials Science, Physics and connecting to cited journals within the same domains and Genetics. Collectively, these pathways reveal convergent-divergent dynamics.

Elsevier-published journals demonstrate predominant influence in the biomaterials field, with multiple titles leading in both publication volume and impact metrics (Table 4). *Acta Biomaterialia* (28 articles, 2023 IF: 9.4) and *Biomaterials* (27 articles, 2023 IF: 12.8) exhibit high research output coupled with exceptional impact factors. Further reinforcing this dominance, *Chemical Engineering Journal* (26 articles, 2023 IF: 13.4) ranks among the top-tier publications in terms of scholarly impact and productivity.

**TABLE 4:** Top 10 prolific journals in hydrogel-based spinal cord injury research.

Rank	Sources	Articles	IF (2023)	Publisher
1	Acta Biomaterialia	28	9.4	Elsevier
2	Biomaterials	27	12.8	Elsevier
3	Chemical Engineering Journal	26	13.4	Elsevier
4	Advanced Healthcare Materials	21	10.0	Wiley
5	ACS Applied Materials and Interfaces	19	8.5	American chemical society (ACS)
6	Biomaterials Science	15	5.8	Royal society of chemistry (RSC)
7	Biomedical Materials	15	3.9	IOP publishing
8	International Journal of Biological Macromolecules	15	7.7	Elsevier
9	Journal of Biomedical Materials Research Part A	15	3.9	Wiley
10	Scientific Reports	14	3.8	Springer nature

### 3.5 Analysis of co-occurring keywords and burst terms

**FIGURE 6:**
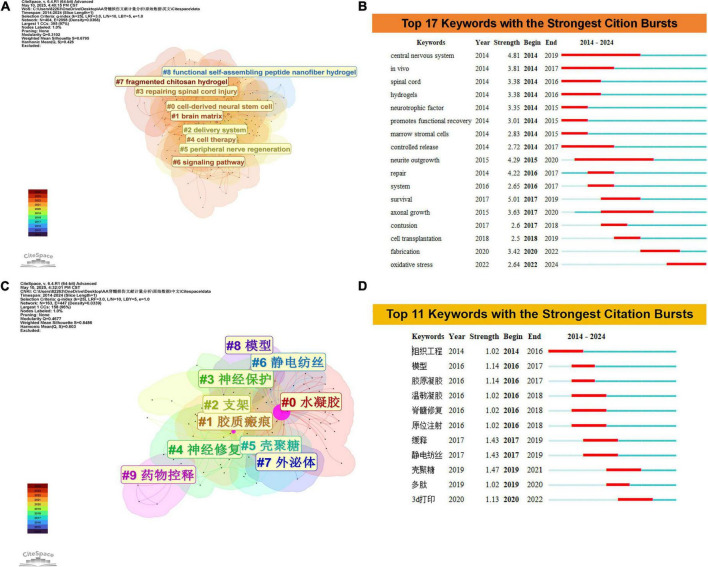
**(A)** Clustering analysis of keyword co-occurrence networks in WoSCC. **(B)** Top 17 keywords with the strongest citation bursts based on WoSCC. **(C)** Clustering analysis of keyword co-occurrence networks in CNKI. **(D)** Top 11 keywords with the strongest citation bursts based on CNKI.

Keyword clustering and burst analysis deconstruct the research field through spatial-temporal dimensions, enabling macrostructural governance and microevolutionary tracking. As illustrated in Figures 6A,B, the developmental trajectory progresses through three phases. The foundational stage (2014–2017) was characterized by burst terms including *central nervous system* (strength 4.81), *in vivo* (3.81), and *spinal cord* (3.38), reflecting neuroanatomical and physiological investigations with animal model validation. Associated Clusters 3 (spinal cord injury repair) and 5 (peripheral nerve regeneration) underscored mechanistic explorations of neural repair. The technology-driven phase (2017–2022) featured burst terms *axonal growth* (3.63) and *fabrication* (3.42), supported by Clusters 8 (self-assembling peptide nanofiber hydrogels) and 7 (chitosan hydrogels), which advanced hydrogel-mediated delivery platforms for neural regeneration. The current emerging phase (2022–2024) is marked by *oxidative stress* (2.64) and Cluster 6: signaling pathways, signaling a transition toward redox imbalance pathophysiology and materials science-molecular biology interdisciplinary integration, thereby establishing targeted therapeutic frameworks. This progression epitomizes a foundational mechanisms → technological innovation → molecular targeting paradigm.

Analysis of the CNKI database reveals a triphasic progression in research hotspots within this field (Figures 6C,D). During the foundational exploration phase (2014–2016), burst terms including “tissue engineering,” “model,” and “collagen gel” correlated with Cluster 8: model development and Cluster 0: hydrogel systems, highlighting prioritized foundational biomaterial construction (e.g., collagen gel fabrication) alongside *in vitro* validation platforms, which established a paradigm for hydrogel property optimization. The technical intensification phase (2016–2019) featured burst terms “sustained-release” (strength = 1.43) and “chitosan” (strength = 1.47), coupled with Clusters 5: chitosan applications and 6: electrospinning techniques, demonstrating advancements in functionalized material systems (e.g., sustained-release carriers) and processing technological innovations (e.g., electrospun scaffolds). Significantly, the burst term “spinal cord repair” propelled Cluster 4: neural repair toward clinical translation, forming a material-technology-therapy translational axis. In the emerging technology phase (2020–2024), the burst term “3D printing” (strength = 1.13) signals the integration of additive manufacturing techniques, marking a strategic shift toward precision biomanufacturing frameworks.

The distinctions in research priorities between the Web of Science (WOS) and CNKI databases manifest through three core dimensions. WOS centers on molecular pathogenesis and interdisciplinary integration, focusing on fundamental mechanisms such as oxidative stress and signaling pathways (e.g., “central nervous system” with burst strength = 4.81). This focus drives the development of advanced materials including self-assembled peptide nanofiber hydrogels, thereby establishing a fundamental-to-molecular targeting paradigm. In contrast, CNKI prioritizes biomaterials engineering and clinical translation, exemplified by collagen gel fabrication and electrospinning optimization (e.g., “chitosan” with burst strength = 1.47). Through its material-technology-treatment multidisciplinary integration framework (e.g., 3D bioprinting), CNKI accelerates the commercialization of implantable neural devices, demonstrating a rapid technological iteration-clinical validation translational cycle. Collectively, these databases propel neural regeneration through complementary approaches—WOS via scientific depth and CNKI through engineering scalability.

### 3.6 Analysis of co-cited references

Co-citation analysis, a pivotal method in bibliometrics, investigates the relationships and knowledge structures among references by examining how two publications are simultaneously cited by one or more subsequent works. The core premise posits that co-cited references exhibit thematic or contextual connections, with stronger associations indicated by higher co-citation frequencies. Table 5 identifies the top five co-cited references: the most frequently co-cited is a motor function rating scale for rats, followed by a study on biomimetic three-dimensional-printed scaffolds for spinal cord injury repair, a publication addressing traumatic spinal cord injuries, and two articles investigating stem cell-mediated repair mechanisms for spinal cord injuries.

**TABLE 5:** Top cited references in hydrogel and spinal cord injury research.

Rank	Citations	Author	Cited references (WoSCC)	Journal	Year	IF (2023)
1	110	Basso DM	A sensitive and reliable locomotor rating scale for open field testing in rats	Journal of neurotrauma	1995	3.9
2	94	Koffler J	Biomimetic 3D-printed scaffolds for spinal cord injury repair	Nature medicine	2019	58.7
3	91	Ahuja CS	Traumatic spinal cord injury	Nature reviews disease primers	2017	79.0
4	72	Assinck P	Cell transplantation therapy for spinal cord injury	Nature neuroscience	2017	21.3
5	68	Fan L	Directing induced pluripotent stem cell derived neural stem cell fate with a three-dimensional biomimetic hydrogel for spinal cord injury repair	ACS Applied materials and interfaces	2018	8.5

**FIGURE 7:**
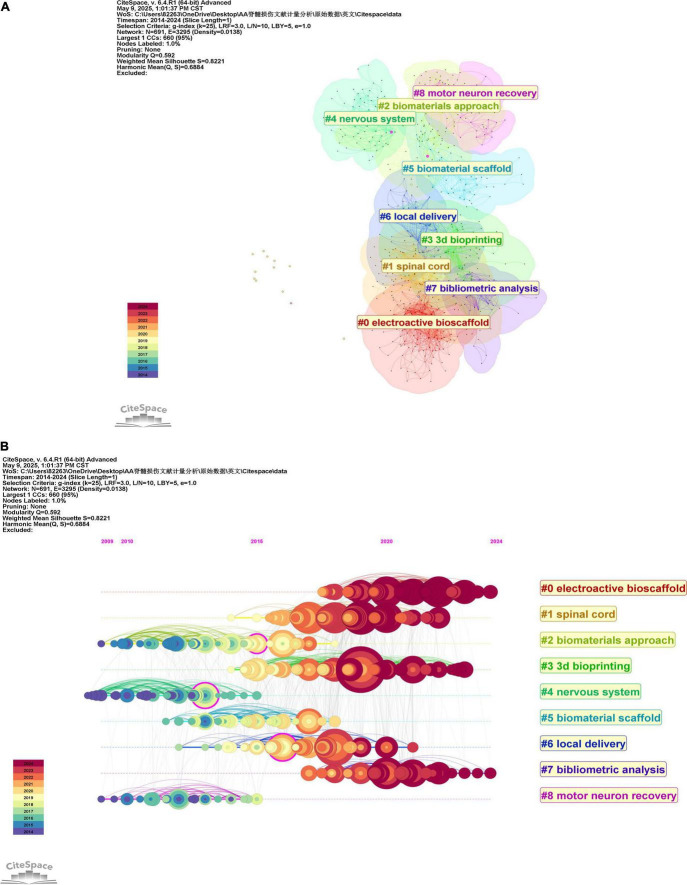
**(A)** CiteSpace visualization of co-cited literature clustering **(B)** Timeline view of these co-cited reference clusters.

This study investigates the application of hydrogels in spinal cord injury through bibliometric clustering analysis and reveals core research directions (Figure 7A): material development (#0 electroactive bioscaffold, #2 biomaterials approach, #5 biomaterial scaffold), technological applications (#3 3dbioprinting), mechanistic integration (#1 spinal cord, #4 neural system), therapeutic strategies (#6 local delivery), and efficacy validation (#8 motor neuron recovery), complemented by domain analysis (#7 bibliometric analysis).

The literature is chronologically arranged from left to right along a horizontal timeline (2014–2024), with annotated temporal nodes to visualize the emergence, persistence, and decline of research themes. Larger nodes denote high-impact publications, while smaller nodes represent marginal studies, color-coded to distinguish different time periods (Figure 7B). Evidently, the leftmost clusters (#2 biomaterials approach, #4 neural system, and #8 motor neuron recovery) correspond to foundational themes in the early research phase. Conversely, clusters #0 electroactive bioscaffold, #3 3d bioprinting, and #7 bibliometric analysis mark current emerging research priorities in this field.

### 3.7 Analysis of research focus coverage

**FIGURE 8:**
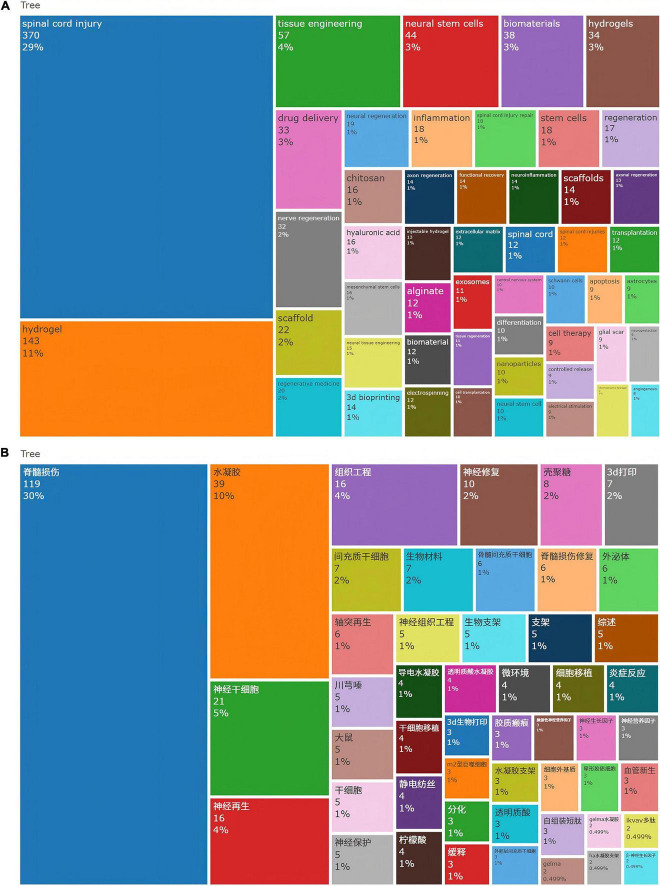
**(A)** Top 50 keywords in the field of hydrogels and spinal cord injury based on WoSCC **(B)** Top 50 keywords in the field of hydrogels and spinal cord injury based on CNKI.

Based on high-frequency term analysis, current research in SCI repair focuses on SCI (*n* = 370) as the primary pathological model. Leveraging tissue engineering (*n* = 57) and regenerative medicine (*n* = 20) frameworks, studies prioritize developing hydrogel-based systems (e.g., chitosan, hyaluronic acid, alginate). These are combined with 3D bioprinting (*n* = 14) and electrospinning (*n* = 12) to fabricate biomimetic scaffolds (*n* = 36) replicating the extracellular matrix (*n* = 12) microenvironment. Key strategies include: (1) Cell therapy (*n* = 9): Transplantation of neural stem cells (*n* = 44), mesenchymal stem cells (*n* = 16), and Schwann cells (*n* = 10) to drive neural and axonal regeneration (cumulative *n* = 78);(2) Drug delivery systems (*n* = 33): Utilizing nanoparticles (*n* = 10) for controlled release (*n* = 9) to modulate neuroinflammation/inflammation (cumulative *n* = 32), suppress glial scar formation (*n* = 9), and enhance neuroprotection (*n* = 9); (3) Microenvironment modulation: Synergistically promoting angiogenesis (*n* = 8) and functional recovery (*n* = 14). The field integrates advanced technologies such as injectable/thermosensitive hydrogels (cumulative *n* = 22), exosomes (*n* = 11), and electrical stimulation (*n* = 9), aimed at overcoming SCI microenvironmental barriers to achieve structural repair and functional reconstruction (Figure 8A).

Following normalization of categorized frequency data, systematic disparities emerge between Chinese and English research: Chinese publications demonstrate pronounced emphasis on hydrogel technologies (constituting 20.7% of total frequency vs. 15.9% in English counterparts), yet exhibit imprecise terminology for technical specifications (e.g., “3D printing” without distinguishing biomanufacturing applications). Conversely, English research establishes comprehensive therapeutic loops through predominant focus on drug delivery systems (4.4% vs. 1.4% in Chinese studies), functional recovery evaluation (1.2% vs. 0%), and neuroimmune modulation (2.7% vs. 1.8%). Regarding indigenous specialization, Chinese research highlights traditional pharmacopeia (e.g., Chuanxiongzine at 2.3%) and stem cell transplantation (9.5% vs. 3.4% in English literature) (Figure 8B); while in mechanistic depth, English studies leverage semantically integrated terminology to achieve multidisciplinary synthesis, revealing greater potential for improvement in Chinese research regarding innovation chains and systemic pathophysiological exploration.

## 4 Discussion

### 4.1 General information and knowledge base

Global hydrogel-SCI research exhibits divergent trajectories: CNKI publications peaked in 2022 then declined, while WoSCC output shows sustained growth (> 15%/yr) with quadratically accelerating citations (*R*^2^ = 0.9989) (Figures 2A,B), confirming ecosystem maturation. Bibliometric analysis also reveals a bimodal global research ecosystem jointly driven by China and the United States, collectively accounting for 76.2% of total academic output. While China leads in publication volume (382 articles) and aggregate citation count (12,214 citations), the United States demonstrates superior research quality metrics, evidenced by its significantly higher citations per article (52.3 vs. China’s 32.0) (Table 1). This dichotomy underscores both complementary strengths and competitive dynamics in advancing hydrogel technologies for spinal cord injury rehabilitation, with China’s scale-driven productivity complementing U.S. leadership in high-impact innovation.

Beyond disparities in publication volume, fundamental structural divergences exist between WoSCC and CNKI repositories, rooted in differential innovation ecosystems: WoSCC embodies mechanism-driven innovation chains (basic → translational → evaluative research), maintaining supremacy in systemic therapeutics (e.g., delivery-immunity-functional recovery continuum) through standardized terminology and international collaboration (Figure 8A). Conversely, CNKI leverages resource-driven technical applications (indigenous pharmacopeia + clinical stem cell expertise), yet exhibits developmental gaps in interdisciplinary integration and mechanistic depth due to insufficient terminology standardization and unidimensional evaluation frameworks (Figure 8B). Consequently, Chinese research requires establishing multidisciplinary consortia, enhancing functional evaluation systems (e.g., incorporating “neurological functional recovery” as standardized keyword), and promoting diagnostic-targeted lexical harmonization (e.g., explicit use of “3D bioprinting” terminology) to bridge innovation chain discontinuities.

Hydrogel research in the field of SCI exhibits significant geographical concentration and interdisciplinary integration. Chinese institutions, such as Zhejiang University and the Chinese Academy of Sciences, dominate global rankings of high-productivity organizations (based on WOSCC and CNKI data). However, institutional collaboration networks reveal robust domestic partnerships but limited international engagement (Figure 5A). High-impact authors (e.g., XIAO JIAN, DAI JIANWU) demonstrate academic leadership through notable h-index and citation metrics, particularly DAI JIANWU, whose rapid ascent in g/m-indices since 2020 reflects accelerated breakthroughs by emerging scholars through high-quality research (Table 3). Journal co-citation analysis further delineates knowledge dissemination patterns: core journals (e.g., BIOMATERIALS) anchor research paradigms via high link strength, while the “convergence-divergence” trajectory in dual-map overlays (Figure 6B) illustrates thematic penetration from materials chemistry into neurobiology and clinical medicine, underscoring the interdisciplinary-driven nature of hydrogel technology.

Visual analysis of keywords and co-cited references revealed that both WOS and CNKI have a greater interest in 3D bioprinting and electroactive bioscaffolds.

### 4.2 Research hotspots and prospects

#### 4.2.1 Oxidative stress and antioxidant hydrogel systems

Our bibliometric analysis reveals that oxidative stress, as a research hotspot at the molecular mechanism level, demonstrates significantly greater depth of discussion and higher frequency of occurrence in WoSCC literature compared to CNKI literature. This reflects the international research community’s stronger emphasis on exploring fundamental pathological mechanisms. Oxidative stress is a critical driver of secondary injury processes following SCI. The mechanisms involved are multifaceted and interconnected (Jia et al., 2011; Tran et al., 2018).

Oxidative stress functions as a core pathological driver in secondary SCI, mediated by excessive reactive oxygen species (ROS) production and compromised antioxidant defenses that collectively induce neuronal/glial degeneration (Ashok et al., 2022). Mitochondrial dysfunction—particularly impaired electron transport chain activity—generates pathological levels of superoxide anion (O_2_^–^), hydrogen peroxide (H_2_O_2_), and hydroxyl radicals (⋅OH) at lesion sites (Battogtokh et al., 2018).

ROS propagate multimodal cellular damage through three synergistic mechanisms: preferential oxidation of mitochondrial membrane lipids initiates autocatalytic peroxidation cascades; oxidative modifications compromise protein integrity; and DNA strand breaks occur, collectively triggering mitochondrial depolarization and MLKL-dependent necroptosis via RIPK1/RIPK3 pathway activation (Blaser et al., 2016; Fan et al., 2018; Tian et al., 2020; Stojkovic et al., 2024).

Antioxidant system collapse manifests through Nrf2-ARE pathway dysregulation, reduced glutathione (GSH) depletion, and compromised superoxide dismutase/catalase activities (Ballatori et al., 2009; Sun et al., 2022). Such failure critically impairs ROS detoxification capacity, enabling persistent oxidative damage.

A self-amplifying inflammatory-oxidative loop emerges: ROS activate NF-κB signaling, promoting TNF-α, IL-1β, and IL-6 release that recruits M1-polarized macrophages (Li et al., 2022; He et al., 2024). These macrophages further elevate ROS via NADPH oxidase, reactivating NF-κB signaling while suppressing neural stem cell differentiation and axonal regeneration—thereby perpetuating tissue destruction (Qin et al., 2004; Wang and He, 2022).

Hydrogel-Mediated Antioxidant Strategies: Current research focuses on smart hydrogel systems loaded with antioxidants designed to targetedly modulate the oxidative microenvironment within the lesion site via sustained release properties (Hu et al., 2024). For instance, ROS-responsive hydrogels sense the burst of ROS at the injury site and dynamically release superoxide dismutase mimetics (e.g., MnTBAP), enabling simultaneous scavenging of multiple free radicals (O_2_^–^, H_2_O_2_, ⋅OH) (Li et al., 2023). These advanced systems exhibit significant superiority over conventional systemic drug administration. Their enhanced local enrichment capacity effectively suppresses the activation of the NF-κB inflammatory pathway, Leading to a reduction in gliotic scar thickness (Zhou et al., 2016). Beyond ROS-responsive systems, hydrogels also achieve temporally controlled release through pH-responsive mechanisms (Cai et al., 2023). pH-responsive hydrogels utilize ionizable groups (e.g., carboxyl/amino) that undergo protonation/deprotonation to sense pH fluctuations, maintaining stability at neutral pH while triggering conformational shifts and targeted drug release in acidic inflammatory environments like SCI sites, as demonstrated by Rogina et al.’s chitosan-hydroxyapatite hydrogel which employs NaHCO3 for rapid *in situ* gelation (< 4 min) and acid-responsive therapeutic molecule release (Rogina et al., 2017).

#### 4.2.2 3D bioprinting technology and 4D materials

A stark generational disparity characterizes 3D printing applications in SCI research across linguistic corpora: WoSCC-indexed studies universally adopt standardized terminology “3D bioprinting” (100% occurrence), strictly delimiting biomedical contexts. Conversely, CNKI publications exhibit terminological fragmentation, with 70% utilizing the industrially connotated “3D printing” while only 30% precisely deploy “3D bioprinting.” Technologically, WoSCC prioritizes advanced biofunctionalization (e.g., integration of bioprinting with electroconductive biointerface engineering), whereas CNKI concentrates on elementary fabrication (e.g., mechanical optimization of hydrogel scaffolds). Crucially, WoSCC constructs complete therapeutic translation pathways—scaffold bioprinting → engineered bioactivation → functional recovery validation (quantitatively linked to electrophysiological/behavioral metrics)—while CNKI remains operationalized through preliminary manufacturing phases (e.g., *in vivo* implantation) without systemic incorporation of biological performance benchmarks or functional efficacy continua.

3D bioprinting integrates additive manufacturing, biomaterials, and cell biology to fabricate functional neural constructs through layer-by-layer deposition of biocompatible materials and living cells (Song et al., 2022; Kim et al., 2023). By enabling bioinspired scaffold design, multicellular patterning, and stimuli-responsive functionalization, this technology overcomes static limitations of conventional scaffolds, offering multi-scale regenerative strategies for spinal cord injury (SCI) repair (Joung et al., 2018; Koffler et al., 2019). Key advancements include: (1) Bioinspired Scaffold Guidance: Extrusion-bioprinted linear hydrogel microchannels enhance axonal elongation and myelination via architectural alignment (Joung et al., 2018). Photolithographic multi-channel scaffolds direct neural progenitor differentiation to restore electrophysiological signaling (Yuan et al., 2022); (2) Multicellular Synergy: 3D-bioprinted porous scaffolds co-encapsulating neural stem cells and oligodendrocytes sustain neurotrophic factor release for axonal regrowth (Liu S. et al., 2022). Fibrin scaffolds with induced pluripotent stem cells enable directional motor neuron differentiation while mitigating secondary injury (De la Vega et al., 2018). (3) Electroactive Microenvironments: Polycaprolactone/polypyrrole conductive scaffolds drive Schwann cell differentiation and neurite outgrowth (Entezari et al., 2022). Multi-walled carbon nanotube scaffolds triple axonal density and restore motor function in nerve defect models through combined topographical/electrical cues (Lee et al., 2018).

While 3D bioprinting enables precise spatial control of scaffold architecture, emerging 4D bioprinting extends this capability by incorporating stimuli-responsive hydrogels that dynamically adapt to the spinal cord microenvironment post-implantation (El-Husseiny et al., 2023). This temporal dimension addresses a critical limitation of static scaffolds—their inability to evolve with injury progression (Sadeghzade et al., 2022). While research on 4D bioprinting for SCI remains relatively scarce, 4D-responsive materials are advancing rapidly in regenerative medicine applications. For instance, Chiang et al. developed a hierarchical hydrogel system that enables 4D spatiotemporal control of biomolecule distribution within anisotropic microcorrugated topographies via electromanipulation of microcapsules. This technology leverages the precise release capability of microcapsules synergistically with the directional topological architecture of hydrogels, providing an innovative platform for spinal cord regeneration (Chiang et al., 2021).

#### 4.2.3 Electroactive bioscaffolds

Electroactive scaffold research manifests a generational divide between “static material engineering” (CNKI) and “dynamic neural interfaces” (WoSCC)—the former prioritizes conductive property enhancement (disproportionately high representation yet insufficient technical depth), while the latter achieves neural functional restoration through coupled programmable electrical stimulation, immunomodulation, and neural remodeling.

Electroactive bioscaffolds integrate conductive components to regulate cellular behaviors through electrical signal transduction in SCI repair (Palza et al., 2019), categorized as: (1) Conductive scaffolds: Poly(3,4-ethylenedioxythiophene)/lignin composites with neural-tissue conductivity (0.60 S/m) direct neural differentiation (Gao et al., 2023); Injectable hydrogels enable neuronal commitment/glial scar suppression via electroactive interfaces (Liu S. et al., 2022). (2) Electroresponsive systems: Capacitive coupling hydrogels generate endogenous microfields via electromagnetic induction (5 MHz), enhancing axonal regrowth non-invasively (Wu et al., 2024). (3) Drug-delivery platforms: Electrospun scaffolds provide dual structural/biological signaling control (Ji et al., 2010).

Electrical signal transduction restores neuronal resting potential (−70 mV) via exogenous stimulation, activating voltage-gated Na^+^/K^+^ channels (Ferrigno et al., 2020). Subsequent voltage-gated calcium channel (VGCC) activation elevates intracellular Ca^2+^, triggering calcium/calmodulin-dependent kinase II (CaMKII)-dependent axonal regeneration (Jara et al., 2020; Gordon, 2024). Concurrently, aligned nanofibers (e.g., electrospun poly-L-lactic acid (PLLA) fibers) facilitate contact-guided Schwann cell migration to form axonal support structures (Behtaj et al., 2022), while electrostimulation upregulates brain-derived neurotrophic factor (BDNF)/neurotrophin-3 (NT-3)-tropomyosin receptor kinase B (TrkB) signaling, activating phosphatidylinositol 3-kinase/protein kinase B (PI3K/Akt) and mitogen-activated protein kinase (MAPK) pathways to enhance neuroplasticity (Ni et al., 2023). Parallel MAPK/extracellular signal-regulated kinase (ERK) and PI3K/Akt activation drives angiogenesis (Katoh, 2023), synergistically with scaffold-released anti-inflammatory factors that suppress microglial M1 polarization to optimize the regenerative niche (Wang et al., 2021).

#### 4.2.4 Evidence-based coordination strategies

Based on the thematic disparities identified between Chinese and English literature (sections 3.5 and 3.7), we propose the following evidence-driven alignment strategies to optimize global research synergy: First, leverage CNKI’s strength in applied hydrogel engineering (e.g., sustained-release systems) and WoSCC’s dominance in molecular pathology (e.g., oxidative stress, signaling pathways) to co-develop mechanism-guided functional hydrogels. For example, embed WoSCC-validated antioxidant agents (e.g., quercetin) into CNKI-optimized chitosan microspheres for targeted ROS scavenging. Second, combine WoSCC’s systemic therapeutic frameworks (e.g., neuroimmunity-functional recovery loops) with CNKI’s clinical trial expertise in stem cell-hydrogel integration to accelerate multicentric validation of biomaterial efficacy.

### 4.3 Limitations

Although bibliometrics and literature visualization analysis can reveal research trends, they exhibit significant limitations: data constraints stem from coverage bias and update delays in mainstream databases; quantitative metrics like citation frequency are susceptible to self-citation manipulation and fail to distinguish research quality; subjective parameter settings lead to clustering discrepancies; inadequate identification of interdisciplinary studies and emerging fields; and risks of artificial hotspot manipulation and academic distortion due to overreliance on metrics. These issues necessitate multi-source data validation and expert qualitative assessment to enhance analytical validity.

Although this study utilized both the Web of WoSCC and CNKI databases for cross-comparative analysis and mutual supplementation, three fundamental errors persist in cross-database comparisons: (1) terminological discrepancies (e.g., semantic divergences between Chinese and English keywords leading to literature retrieval omissions); (2) metric system incompatibility (e.g., non-equivalence between journal impact factors and Chinese core journal evaluation criteria); (3) structural data conflicts (e.g., parsing failures in CNKI journal, institutional, and reference analyses due to formatting inconsistencies).

## 5 Conclusion

Bibliometric analysis has revealed rapid global advancements in hydrogel and SCI research, demonstrating bipolar leadership between China and the United States. Despite divergent publication patterns in China, marked by declining CNKI-based domestic output and sustained international growth indexed in the WoSCC, Zhejiang University has achieved breakthroughs and has published the majority of its research outputs. Notably, keyword co-occurrence and co-cited references further identify current research frontiers, including spinal-cord-injury pathological mechanisms (e.g., oxidative stress), 3D bioprinting, and electroactive bios scaffolds. Building on current research priorities, this study explores advanced applications of stimuli-responsive hydrogels (e.g., ROS-responsive mechanisms and pH-triggered strategies) and 4D materials for SCI regeneration.

## Data Availability

The original contributions presented in the study are included in the article/supplementary material, further inquiries can be directed to the corresponding author.
